# Kinin B1 Receptor Mediates Bidirectional Interaction between Neuroinflammation and Oxidative Stress

**DOI:** 10.3390/antiox12010150

**Published:** 2023-01-08

**Authors:** Drew Theobald, Srinivas Sriramula

**Affiliations:** Department of Pharmacology and Toxicology, Brody School of Medicine at East Carolina University, Greenville, NC 27834, USA

**Keywords:** oxidative stress, hypertension, kinin B1 receptor, neurons, neuroinflammation

## Abstract

Hypertension is associated with increased expression of kinin B1 receptors (B1R) and increased levels of pro-inflammatory cytokines within the neurons. We previously reported that angiotensin II (Ang II) upregulates B1R expression and can induce neuroinflammation and oxidative stress in primary hypothalamic neurons. However, the order in which B1R activation, neuroinflammation, and oxidative stress occur has not yet been studied. Using primary hypothalamic neurons from neonatal mice, we show that tumor necrosis factor (TNF), lipopolysaccharides (LPS), and hydrogen peroxide (H_2_O_2_) can upregulate B1R expression and increase oxidative stress. Furthermore, our study shows that B1R blockade with R715, a specific B1R antagonist, can attenuate these effects. To further confirm our findings, we used a deoxycorticosterone acetate (DOCA)-salt model of hypertension to show that oxidative stress is upregulated in the hypothalamic paraventricular nucleus (PVN) of the brain. Together, these data provide novel evidence that relationship between oxidative stress, neuroinflammation, and B1R upregulation in the brain is bidirectional, and that B1R antagonism may have beneficial effects on neuroinflammation and oxidative stress in various disease pathologies.

## 1. Introduction

Hypertension has been found to be associated with increased activity of the kallikrein–kinin system (KKS), which leads to vasoconstriction and inflammation [1,2]. The KKS belongs to a family of vasoactive pro-inflammatory peptides that play a part in regulating blood pressure and inflammation and are primarily known as bradykinin and kallidin [3]. Bradykinin and its active metabolite des-arg^9^-bradykinin mediate their physiological effects through two G-protein coupled receptor subtypes, kinin B1 (B1R) and B2 (B2R) receptors [3,4,5]. B2R is constitutively expressed, whereas B1R expression is relatively low at physiological conditions and can be upregulated in the presence of inflammation and oxidative stress [3,6]. In addition to inflammation and oxidative stress, B1R expression can be induced by its own agonists, des-Arg⁹-BK (DABK) or des-Arg¹⁰-KD (DAKD) [6,7]. It is known that both B1R and B2R are expressed in neurons, and that B1R is more specifically expressed in the human thalamus and hypothalamus [8]. Previously, we showed that neurogenic hypertension is associated with an increased expression of B1R in the hypothalamic paraventricular nucleus (PVN) in a model of DOCA-salt hypertension [1].

Hypertension remains the dominant risk factor for cardiovascular disease, which is the leading cause of death worldwide, despite the implications of lifestyle modifications and readily available hypertension medications [9,10,11]. It is now well established that hypertension is a low-grade inflammatory condition that is induced via increased sympathetic drive, elevated oxidative stress, and an increased release of proinflammatory cytokines within the cardiovascular regulatory regions of the brain, such as the PVN [1,12,13]. Within these cardiovascular regulatory regions, neuroinflammation is known to be the primary mediator of the pathophysiology of many cardiovascular diseases such as hypertension [6,13,14]. There is a bidirectional interaction between the nervous system and the immune system and, as a result, there is an inflammatory response within the nervous system that leads to the activation of proinflammatory cytokines and reactive oxygen species (ROS) that can worsen a disease state [6,15,16,17]. Since inflammatory cells are thought to be a large source of ROS, there is also a bidirectional relationship between the immune system and reactive oxygen species and this relationship regulates the hypertensive response and ultimate end-organ damage [15,18,19].

It is known that B1R is inducible by inflammation, injury, DABK or DAKD, inflammatory cytokines, and bacterial endotoxins [2,6]. Previously, we were able to show that Lys-[des-Arg^9^]-Bradykinin (LDABK) and angiotensin II (Ang II) upregulate B1R expression in primary hypothalamic neurons [20,21]. We also showed that Ang II can increase oxidative stress through ROS production, and that this increase is attenuated using the specific B1R antagonist R715, but not by the specific B2R antagonist HOE [20]. Ang II is also able to increase Nox2 and Nox4 gene expression as well as TNF, IL-6 and IL-1β gene expression, indicating that Ang II can induce neuroinflammation and oxidative stress in primary hypothalamic neurons [20]. These studies suggest that Ang II-induced B1R activation may increase neuroinflammation and oxidative stress in neurons. However, there have been no previous studies in neurons providing direct evidence for which comes first, B1R activation or neuroinflammation and oxidative stress. Therefore, in this present study we first determined the role of TNF (inflammatory cytokine), LPS (bacterial endotoxin), and hydrogen peroxide (ROS) on the activation of B1R using mouse primary hypothalamic neuronal cultures, and then investigated whether B1R activation stimulates oxidative stress or neuroinflammation, or if neuroinflammation or oxidative stress stimulates B1R activation using B1R blockade.

## 2. Materials and Methods

### 2.1. Animals

Experiments were conducted using adult (12–16 weeks old) wildtype (WT) male C57Bl/6NJ mice purchased from Jackson Laboratory and housed in a humidity- and temperature-controlled facility following a 12-h light/dark cycle. Mice were fed standard mouse chow and water ad libitum. All animal studies were approved by the East Carolina University Animal Care and Use Committee (AUP #W254) and were performed per the National Institutes of Health Guidelines for the Care and Use of Laboratory Animals. Mice underwent a DOCA-salt hypertension paradigm as described previously [1]. Mice were briefly anesthetized with isoflurane (2%) and oxygen flow 1 L/min and placed on a heating pad to maintain body temperature. The mice underwent a uninephrectomy in which the right kidney was removed by making an incision to the skin in the retroperitoneal region. Mice were divided randomly into 4 groups (n = 12/group) and were then implanted subcutaneously with a DOCA-silicone sheet (DOCA group, DOCA:silicone = 1:3, DOCA 1 mg/g body weight) or an empty silicone sheet (Sham group). Pre- and post-operative care included buprenorphine injection to relieve pain (0.05 mg/kg, sc). The DOCA group were switched to 1% NaCl in drinking water and the sham group received autoclaved tap water. After the duration of the protocol, mice received heavy anesthesia using 5% isoflurane and were euthanized by decapitation. The brains were collected and stored at −80 °C until further use.

### 2.2. Primary Hypothalamic Neuron Isolation

Primary hypothalamic neurons were cultured and maintained from neonatal mice pups as described in previous studies [20,21,22]. The experimental protocols used for breeding mice were approved by East Carolina University Animal Care and Use Committee (AUP #W261) and were performed in accordance with the National Institutes of Health Guidelines for the Care and Use of Laboratory Animals. Mouse pups were deeply anesthetized using isoflurane (4%) in an oxygen flow (1 L/min) prior to decapitation. Brains were collected and hypothalamic tissue was dissected in Hank’s balanced salt solution (HBSS) (Gibco 14,175-079) then minced into small pieces using a sterile blade. The minced tissue was rinsed twice with HBSS and then was digested in a 15 mL conical tube with HBSS containing 1% trypsin (T1426 Sigma-Aldrich, St. Louis, MO, USA) and 1.5 kU/mL DNaseI (D5025 Sigma Aldrich) for 10 min at 37 °C. Following dissociation, tissue was washed twice using HBSS containing 20% FBS and then twice using HBSS. Using HBSS containing DNaseI, the tissue was further homogenized using a pipette with a 1 mL pipette tip (six times) followed by using a 200 μL pipette tip (six times) attached to a 10 mL serological pipette. After the dissociation, the cells were spun down by centrifugation and the cell pellet was collected and resuspended in complete Neurobasal culture medium supplemented with 2% B27, 0.5 mM GlutaMax, and penicillin/streptomycin (100 U/mL and 100 μg/mL) (Gibco). At a density of 50,000 cells per mL, neurons were plated into poly-L-lysine-coated cell culture plates and grown in a humidified atmosphere of 5% CO2–95% air at 37 °C. On the fourth day of culture, neurons were treated with cytosine arabinofuranoside (Ara-C, 2 μM, Sigma Aldrich C1768) to suppress non-neuronal cell proliferation. Hypothalamic primary neurons were cultured for at least 10 days prior to experimentation. Treatment duration and doses of LDABK (300 nM), R715 (10 μM), SR (10 μM), H_2_O_2_ (10 μM), TNF (10 ng), and LPS (100 ng) were based on our preliminary studies and the published literature [6,22]. All drug treatments were dissolved in sterile PBS as a vehicle control. LPS purified from Escherichia coli O55:B5 (Sigma L4005, CAS-No. 93572-42-0) was used as it has been previously shown to induce neuroinflammation via the TLR4/CD14-mediated pathway [23,24].

### 2.3. Real Time RT-PCR

Using real time RT-PCR, gene expression was measured as described previously by our lab [22,25]. Using primary hypothalamic neurons treated with either TNF or LPS, total RNA was extracted using a Direct-Zol RNA miniprep plus kit (Zymo Research) according to the manufacturer’s protocol. The concentration of RNA was measured using a spectrophotometer (NanoDrop One). Using a QuantStudio 6 Flex real time PCR machine (Applied Biosystems), RT-PCR amplification was performed with Power SYBR Green RNA-to-CT one step kit (Applied Biosystems). Data were normalized to β-actin expression by the 2^−(ΔΔCT)^ comparative method and expressed as a fold change compared to control.

### 2.4. Immunofluorescence Staining

Once neurons were cultured for 10 days on poly-L-lysine-coated glass cover slips in 12-well plates and treated with various agonists for time points, they were fixed with 4% paraformaldehyde for 15 min. The neurons were rinsed three times for 5 min using 1 X PBS followed by permeabilization with 0.1% Triton X-100 in 1 X PBS for 10 min. Cells were blocked with 2.5% donkey serum in 1 X PBS containing 0.2% Tween20 for 1 h before being incubated overnight at 4 °C with appropriate primary antibodies that have been validated in knockout mouse tissues, MAP2 antibody (NBP3-05552, lot 16278-011222, Novus Biologicals, 1:500), or B1R (#ABR-011, lot An-01, Alomone labs, 1:500 dilution) [25]. Following this, the cells were rinsed with 1 X PBS + 0.2% Tween20 three times then incubated with appropriate Alexa Fluor conjugated secondary antibodies (Life Technologies, 1:1000 dilution) for 1 h at room temperature. Cells were washed three times with 1 X PBS + 0.2% Tween20 then mounted onto slides using ProLong Gold antifade reagent with DAPI (Invitrogen). Images were captured using an Echo Revolve Microscope.

### 2.5. Measurement of ROS

Levels of reactive oxygen species (ROS) were identified by dihydroethidium (DHE), which is relatively specific for the measurement of superoxide. DHE will exhibit a blue fluorescence until it is oxidized and interacts with the cell’s DNA to produce a bright fluorescent red staining of the nucleus. Freshly cut brains from DOCA and Sham mice were incubated with 10 μM DHE (Invitrogen) in a light protected humidified chamber at 37 °C for 15 min. Primary neurons that were cultured and treated in 12-well plates on poly-L-lysine glass coverslips were given 10 μM DHE and incubated in a light protected humidified chamber at 37 °C for 15 min. Fluorescent images of the DOCA/Sham brains and the primary neurons were obtained with a fluorescent microscope (Echo Revolve). Mean fluorescence intensity of the image was measured using ImageJ software (NIH) for quantification. To supplement this, production of ROS was also measured by spectrofluorometry using DHE probes. The neurons were cultured in 48-well plates and were briefly treated for their respective group. Following this, 10 μM DHE was added for 15 min. The plate was read via spectrofluorometer (Tecan Infinite m200) and fluorescence was detected at an excitation wavelength of 488 nm and emission wavelength of 610 nm. Data are expressed as total fluorescence (relative fluorescent units, RFU).

### 2.6. Statistics

Statistical analyses were performed using GraphPad Prism 9 (GraphPad Software). Data are presented as mean ± standard error of the mean (SEM). Using one-way analysis of variance followed by Tukey’s multiple comparisons test, multiple comparisons were made, and differences were considered statistically significant at *p* < 0.05.

## 3. Results

### 3.1. B1R Activation Induces Reactive Oxygen Species Production in Primary Hypothalamic Neurons

**Figure 1:**
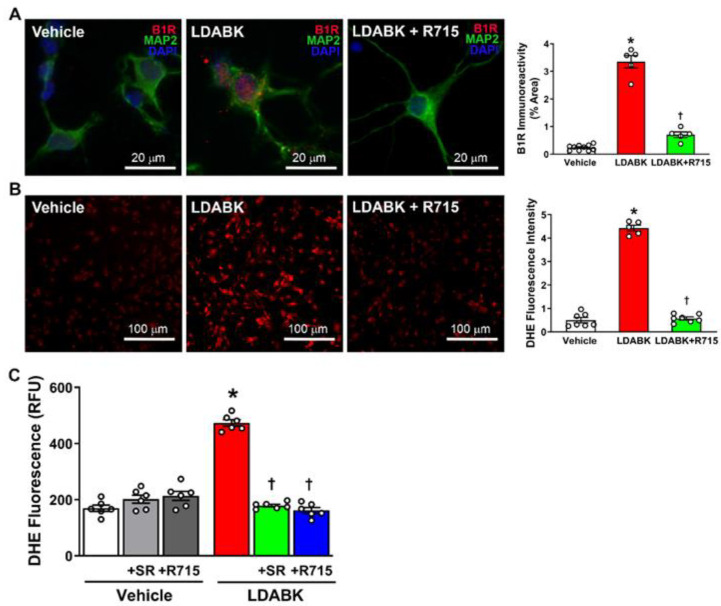
LDABK treatment on primary hypothalamic neurons. (**A**) Immunofluorescence staining and quantification for B1R expression (n = 5–10/group). (**B**) Superoxide generation using dihydroethidium (DHE) staining (n = 5–10/group). (**C**) ROS production measured by microplate DHE assay (n = 6/group). Statistical significance: One-way ANOVA followed by Tukey’s multiple comparisons test. * *p* < 0.05 compared to vehicle. ^†^ *p* < 0.05 compared to LDABK.

To investigate the role of LDABK, a B1R selective agonist, in the production of reactive oxygen species, we used primary neurons cultured from the hypothalamus of neonatal mice and cultured them for 10 days prior to stimulating them with LDABK (300 nM, 24 h) with or without pretreatment using the B1R specific antagonist R715 (10 μM). First, using double immunostaining, we confirmed previous findings that LDABK increases B1R expression [21,22]. As shown in Figure 1A, treatment of the neurons with 300 nM of LDABK increased the expression of B1R. Furthermore, this effect was prevented by 2-h pretreatment with B1R antagonist R715, indicating that LDABK induced B1R activation. Previously, it has been suggested that reactive oxygen species (ROS) play a vital role in the development of neurogenic hypertension [26,27]. Therefore, to determine the role of B1R activation in the production of reactive oxygen species, we used dihydroethidium (DHE) to measure the oxidative potential of the cells. LDABK exposure increased DHE red fluorescence, indicating an increase in the generation of reactive oxygen species. Interestingly, pretreatment with R715 was able to mitigate the increase in production by LDABK, confirming that B1R activation induces ROS generation in hypothalamic neurons (Figure 1B). To further confirm these results, we used a microplate DHE assay and found that stimulation with LDABK produced a significant increase in ROS generation and that this effect was attenuated by pretreatment with a B1R antagonist (Figure 1C). These results provide evidence that B1R activation in primary hypothalamic neurons increases ROS production and that B1R antagonism may attenuate these effects.

### 3.2. TNF Stimulation Increases Expression of B1R and ROS in Primary Hypothalamic Neurons

**Figure 2:**
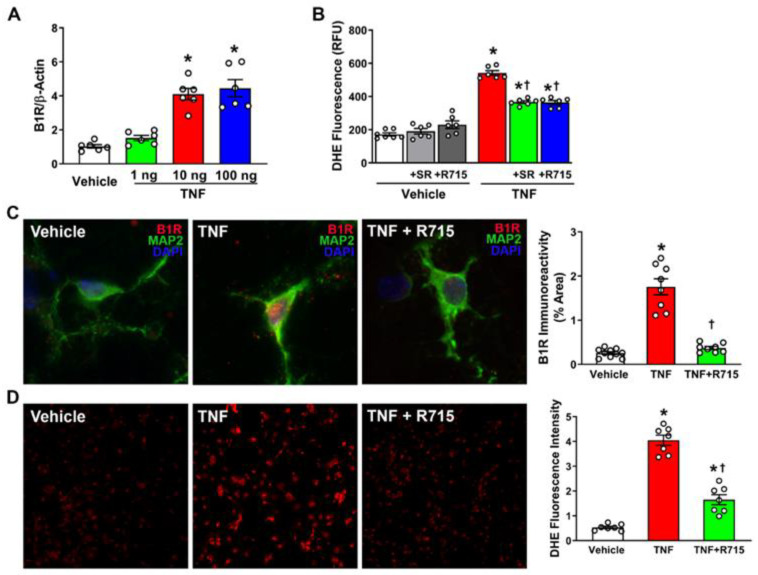
TNF treatment on primary hypothalamic neurons. (**A**) B1R mRNA levels in cultured neurons, measured by real time PCR (n = 6 independent cultures/group). (**B**) ROS generation indicated by microplate DHE assay (n = 6–7/group). (**C**) Quantification and immunofluorescent images of B1R expression (n = 8–10/group). (**D**) ROS production and quantification of DHE staining (n = 7/group). Statistical significance: One-way ANOVA followed by Tukey’s multiple comparisons test. * *p* < 0.05 compared to vehicle. ^†^ *p* < 0.05 compared to TNF.

B1R activation has been shown to be involved in neuroinflammation as well as neurogenic hypertension. Inflammation is an important factor in the pathogenesis of hypertension, therefore we used TNF, a proinflammatory cytokine, to determine whether B1R blockade prevents inflammation-induced B1R expression and oxidative stress. Using RT-PCR, we wanted to determine at what dose TNF induces B1R activation and found that 10 ng TNF significantly increases B1R expression (Figure 2A). Next, we wanted to know if TNF-induced activation of B1R could be mitigated by pretreatment with B1R antagonist R715 and found that pretreatment with R715 was able to attenuate this increase (Figure 2C). Furthermore, we wanted to identify if B1R blockade could prevent inflammation-induced oxidative stress. To determine this, we treated primary hypothalamic neurons with 10 ng TNF with or without 2-h preincubation with 10 μM R715 and used DHE immunofluorescence to identify reactive oxygen species production. The results indicated that preincubation with R715 reduces TNF-induced ROS generation (Figure 2D). To further confirm this, we used a DHE microplate assay and confirmed that B1R antagonism can attenuate TNF-induced ROS and oxidative stress (Figure 2B). Overall, our results indicate that blocking B1R can mitigate inflammation-induced B1R activation and ROS production in primary hypothalamic neurons.

### 3.3. LPS Stimulation Increases Expression of B1R and ROS in Primary Hypothalamic Neurons

**Figure 3:**
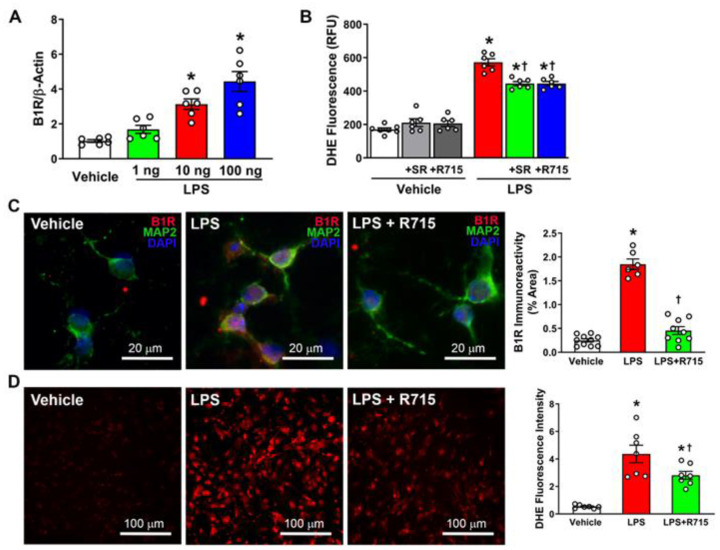
LPS treatment on primary hypothalamic neurons. (**A**) Levels of B1R mRNA measured by real time PCR (n = 6 independent cultures/group). (**B**) ROS production demonstrated using microplate DHE assay (n = 6/group). (**C**) Representative images of B1R expression and quantification (n = 6–10/group). (**D**) Superoxide production and quantification of DHE immunostaining (n = 7/group). Statistical significance: One-way ANOVA followed by Tukey’s multiple comparisons test. * *p* < 0.05 compared to vehicle. ^†^ *p* < 0.05 compared to LPS.

LPS stimulation has been shown to cause the release of inflammatory cytokines in cellular models [28,29]. First, we used RT-PCR to identify at what concentration LPS upregulates B1R and found that 100 ng LPS is the most ideal for activation of B1R (Figure 3A). Following this, we showed that 2-h pretreatment with R715 was able to significantly attenuate LPS-induced B1R expression, as shown by immunofluorescence staining and quantification (Figure 3B). Next, we wanted to identify the role of LPS in reactive oxygen species generation. Following stimulation with LPS with or without B1R blockade, we used DHE immunofluorescence and showed that LPS increases ROS generation, as shown by an increase in red fluorescence (Figure 3D). However, B1R blockade prior to LPS stimulation was able to partially reduce this effect. Furthermore, we followed up with a DHE microplate assay and further confirmed that B1R antagonism attenuates LPS-induced ROS production (Figure 3B). Collectively, these data infer that LPS can activate B1R and induce ROS production, while blocking B1R only partially reduces the effects of LPS stimulation.

### 3.4. B1R Blockade Prevents Hydrogen Peroxide-Induced Oxidative Stress

**Figure 4:**
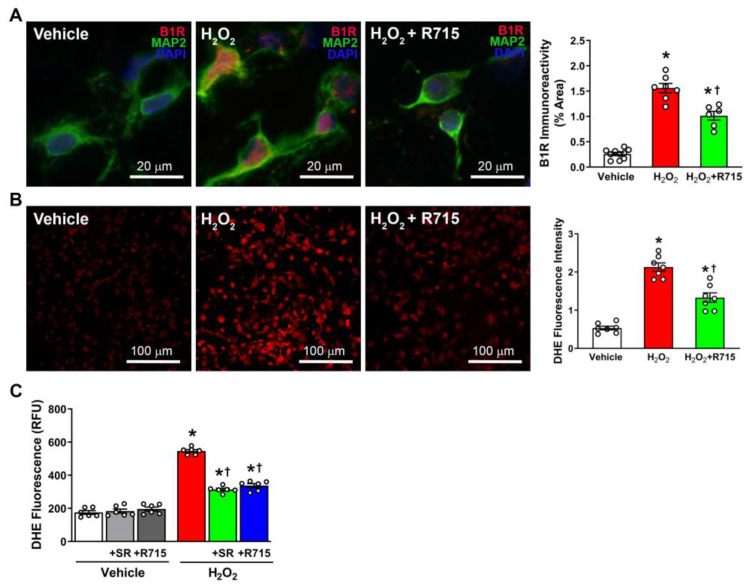
H_2_O_2_ treatment on primary hypothalamic neurons. (**A**) B1R expression indicated by immunofluorescence and quantification (n = 6–10/group). (**B**) Generation of reactive oxygen species using DHE staining (n = 7/group). (**C**) DHE microplate assay revealing ROS generation (n = 6/group). Statistical significance: One-way ANOVA followed by Tukey’s multiple comparisons test. * *p* < 0.05 compared to vehicle. ^†^ *p* < 0.05 compared to H_2_O_2_.

In previous studies, it has been indicated that oxidative stress plays a vital role in the pathogenesis of hypertension [26,27]. Oxidative stress is caused by excessive generation of ROS, so to model this in our neuron cultures, we treated neurons with 10 μM hydrogen peroxide for 24 h. In cellular models, hydrogen peroxide is often used to induce inflammation and oxidative stress [30]. Using immunofluorescence staining, we found that hydrogen peroxide stimulation increases B1R expression, and this increase was mitigated with B1R antagonist R715, indicating that oxidative stress acts, in part, through the B1R pathway (Figure 4A). To further determine the role of B1R in oxidative stress, we used DHE and showed that, as predicted, hydrogen peroxide stimulation increases ROS production. Interestingly, this production was reduced by pretreatment with R715 (Figure 4B). To confirm these findings, we used a microplate DHE assay and once again showed that B1R antagonism prevents hydrogen peroxide-induced oxidative stress (Figure 4C). These results indicate that there is a relationship between B1R activation and oxidative stress, and that B1R blockade may prevent these effects.

### 3.5. DOCA-Salt Hypertension Model Shows Increased ROS Generation

**Figure 5:**
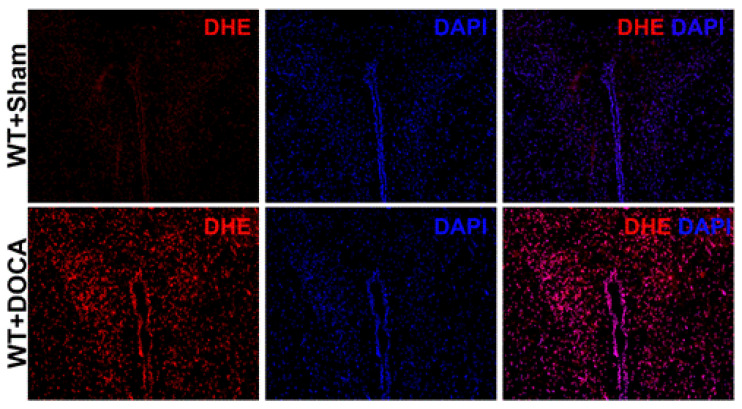
Elevated ROS production in the PVN during DOCA-salt hypertension. Representative images stained for DHE indicating increased ROS production in PVN of WT + DOCA mice compared to WT + Sham mice.

Prior studies have shown that B1R plays a central role in the pathogenesis of DOCA-salt hypertension [1]. To further confirm our findings, we wanted to determine if oxidative stress is increased in DOCA-salt hypertensive mice. Brains from Sham and DOCA mice were cut, using a cryostat, into 10 µm sections and placed onto a slide. Using dihydroethidium immunofluorescence, we showed that ROS generation was increased in DOCA mice compared to Sham, as shown by an increase in red fluorescence (Figure 5). These results further indicate that oxidative stress plays an important role in hypertension.

## 4. Discussion

The kinin B1 receptor is known to be inducible by inflammation, oxidative stress, or injury, however, whether neuroinflammation and oxidative stress precede B1R activation or vice versa was unknown. In this study, we aimed to investigate the interaction between B1R activation on neuroinflammation and oxidative stress. Previous studies have shown that B1R is inducible by its own agonist, LDABK, in hypothalamic neurons [22]. Using immunocytochemistry on primary hypothalamic neurons, we were able to confirm that B1R expression is inducible using its own agonists. Treatment with LDABK induced B1R activation in primary hypothalamic neurons, and this effect was attenuated following pretreatment with B1R antagonists. Previously, we and others have shown that Ang II can upregulate B1R expression and oxidative stress, and that this effect was attenuated using B1R antagonism [20,31,32]. Using dihydroethidium staining and microplate assay, we found that B1R activation by a specific agonist increases superoxide production, and that using a B1R antagonist can blunt this increase in ROS production. These results provide evidence for a direct effect of B1R activation and oxidative stress.

The immune system is of large importance in the pathophysiology of hypertension, and this is primarily due to its effects on elevated inflammation both in the periphery and in the central nervous system [33]. Proinflammatory cytokines such as TNF have been shown to play a vital role in the pathogenesis of cardiovascular diseases, including hypertension [12]. TNF has been shown to modulate the expression of NADPH oxidases, which are a known potential source of ROS within cardiovascular diseases [34]. Previous studies from our lab and others have shown that TNF can induce oxidative stress, leading to cardiac dysfunction and hypertension. On the other hand, TNF blockade, either using gene deficient mice or specific antagonists, can prevent the development of angiotensin II-induced hypertension. Since B1R is also induced by inflammation, whether adverse outcomes with elevated TNF are mediated by B1R activation is not known. In our study, we tested the effect of direct treatment of primary hypothalamic neurons with TNF and measured B1R expression. Our data show that TNF induced B1R expression, as well as increased ROS production. Furthermore, blocking B1R using B1R antagonists R715 or SR was able to reduce the effects of TNF on reactive oxygen species production and B1R activation, indicating that TNF induces ROS production at least partially through a B1R-mediated pathway.

Increasing evidence implicates the role of bacterial endotoxins such as lipopolysaccharides (LPS) in the stimulation of B1R expression [35,36]. LPS have been shown to be a potent stimulator of the innate immune response and are able to initiate the intracellular signaling proteins required to promote cytokine production during the initiation of inflammation [19,37,38]. Furthermore, studies have shown that LPS can increase oxidative stress levels, release proinflammatory cytokines, and can also initiate inflammatory response-mediated hypertension [28,39,40]. However, the role of B1R activation in LPS-induced oxidative stress, specifically in neurons, is relatively unknown. Here, our data confirm that LPS treatment of neurons induced B1R expression and increased ROS production. Additionally, B1R blockade with a specific antagonist was able to attenuate LPS-induced ROS production, suggesting that LPS-mediated effects on oxidative stress are, at least in part, mediated by B1R activation. These data allow us to speculate that LPS may act through a B1R-mediated pathway in the progression of hypertension, and further studies are needed to explore the role of B1R in LPS-induced hypertension.

Oxidative stress is an excessive production of reactive oxygen species, such as superoxide and hydrogen peroxide, and an altered oxidation–reduction state [33]. Generation of oxidative stress within the CNS has been shown to influence systemic inflammation, which alters blood pressure responses [33,41,42,43]. In disease conditions, an imbalance between ROS production and antioxidants that favor ROS production can lead to the disruption of redox signaling and molecular damage. Hydrogen peroxide also acts as a second messenger that has a role in intercellular signaling [44,45]. Using hydrogen peroxide as a stimulant, we wanted to determine the role of oxidative stress in B1R activation and whether it is B1R activation that triggers oxidative stress or if oxidative stress can induce B1R activation. Our data show that hydrogen peroxide stimulation can increase B1R expression in primary neurons, suggesting a direct role of hydrogen peroxide in B1R induction. In addition, hydrogen peroxide treatment increased ROS production, which was blunted by a B1R specific antagonist, suggesting a role of B1R activation in hydrogen peroxide-induced ROS production. These data provide clear evidence that oxidative stress is, in part, mediated through the B1R pathway.

Primary hypothalamic neuronal cultures allow us to use a model with a high resolution compared to immortalized cells, as shown in our previous studies [20,21,22,25]. Our study, to our knowledge, is the first to look at the bidirectional role between B1R activation, neuroinflammation, and oxidative stress in primary hypothalamic neurons, and its role in various disease etiologies. Although we were not able to distinguish the order in which these events occur, we were able to identify the causal role of B1R in oxidative stress and neuroinflammation, further indicating its part in the development of many pathologies, such as hypertension, neurodegenerative diseases, and cardiovascular diseases. We were also able to identify that B1R blockade may serve as an effective agent in reducing neuroinflammation and oxidative stress. Given the pivotal role played by the neuroinflammation and oxidative stress pathways in the progression of cardiovascular and neurodegenerative diseases, we can speculate that B1R targeting therapies could be clinically beneficial. Further studies are needed to help identify which events occur first while also looking at these relationships in other models, such as microglia, neuron–glia cocultures, and in vivo models. Nevertheless, the results of this study provide evidence to support the role of B1R activation in in neurons, and the beneficial effects of B1R blockade on mitigating the effects of neuroinflammation and oxidative stress.

## Data Availability

Data are contained within the article.
